# Overexpression of serine protease HtrA enhances disruption of adherens junctions, paracellular transmigration and type IV secretion of CagA by *Helicobacter pylori*

**DOI:** 10.1186/s13099-017-0189-6

**Published:** 2017-07-25

**Authors:** Aileen Harrer, Manja Boehm, Steffen Backert, Nicole Tegtmeyer

**Affiliations:** Lehrstuhl für Mikrobiologie, Friedrich-Alexander-Universität Erlangen-Nürnberg, Staudtstr. 5, 91058 Erlangen, Germany

**Keywords:** Adherens junction, Tight junction, E-cadherin, *Helicobacter pylori*, Protease, HtrA, Type IV secretion system, T4SS, CagA, EPIYA, Src, Abl, Integrin, Transwell

## Abstract

**Background:**

The serine protease HtrA is an important factor for regulating stress responses and protein quality control in bacteria. In recent studies, we have demonstrated that the gastric pathogen *Helicobacter pylori* can secrete HtrA into the extracellular environment, where it cleaves-off the ectodomain of the tumor suppressor and adherens junction protein E-cadherin on gastric epithelial cells.

**Results:**

E-cadherin cleavage opens cell-to-cell junctions, allowing paracellular transmigration of the bacteria across polarized monolayers of MKN-28 and Caco-2 epithelial cells. However, rapid research progress on HtrA function is mainly hampered by the lack of Δ*htrA* knockout mutants, suggesting that *htrA* may represent an essential gene in *H. pylori*. To circumvent this major handicap and to investigate the role of HtrA further, we overexpressed HtrA by introducing a second functional *htrA* gene copy in the chromosome and studied various virulence properties of the bacteria. The resulting data demonstrate that overexpression of HtrA in *H. pylori* gives rise to elevated rates of HtrA secretion, cleavage of E-cadherin, bacterial transmigration and delivery of the type IV secretion system (T4SS) effector protein CagA into polarized epithelial cells, but did not affect IL-8 chemokine production or the secretion of vacuolating cytotoxin VacA and γ-glutamyl-transpeptidase GGT.

**Conclusions:**

These data provide for the first time genetic evidence in *H. pylori* that HtrA is a novel major virulence factor controlling multiple pathogenic activities of this important microbe.

## Background

*Helicobacter pylori* is a Gram-negative, flagellated pathogen, which persistently colonizes the human stomach [1, 2]. About 50% of the world population carries these bacteria, and infections are associated with chronic, often asymptomatic gastritis in all infected individuals. However, more severe gastric diseases such as peptic ulceration, mucosa-associated lymphoid tissue (MALT) lymphoma and gastric adenocarcinoma can arise in a subset of patients [3, 4]. The clinical outcome of *H. pylori* infection is regulated by several key elements including the genetic predisposition of the host, the bacterial genotype and environmental factors [5–7]. Dozens of bacterial determinants have been described to impact *H. pylori* pathogenicity. Two classical virulence factors are known, the vacuolating cytotoxin (VacA) and the cytotoxin-associated genes pathogenicity island (*cag*PAI). The *cag*PAI encodes a type IV secretion system (T4SS) for transport of the oncoprotein CagA across the bacterial membranes into host target cells [8, 9]. Upon delivery, CagA undergoes phosphorylation at C-terminal Glu-Pro-Ile-Tyr-Ala (EPIYA) sequence repeats by the c-Src and c-Abl family of tyrosine kinases [10–12]. Translocated CagA binds to and activates or inactivates a series of signaling factors in a phosphorylation-dependent and phosphorylation-independent fashion [13, 14]. The T4SS can also induce profound pro-inflammatory responses such as the release of chemokine interleukin-8 (IL-8) via transcription factor NF-κB, which proceeds widely independently of CagA delivery [15–17]. On the other hand, VacA is an autotransporter and secreted into the extracellular space, where it induces multiple responses including cell vacuolation, alteration of endo-lysosomal trafficking, immune cell inhibition and apoptosis [5, 18]. Other pathogenicity-associated processes comprise urease-triggered neutralization of acidic pH, flagella-mediated motility, expression of multiple adhesins (BabA/B, SabA, AlpA/B, HopQ, HopZ, OipA and others), inhibition of T cell proliferation by secreted γ-glutamyl-transpeptidase GGT, and secretion of proteases such as HtrA [3, 19–21].

High temperature requirement protein A (HtrA ) family members comprise a set of evolutionarily related serine proteases and chaperones, which are found in most prokaryotes and eukaryotes [22–24]. HtrA proteases are generally transported into the periplasm, where they form proteolytically active oligomers with important function in protein quality control [25, 26]. Its chief role is to remove damaged, misfolded or mislocalized proteins in the periplasm. HtrA proteins contain no regulatory components or ATP binding domains [22]. Thus, they are commonly referred to as ATP-independent chaperone-proteases. Bacterial HtrA proteases commonly comprise an N-terminal signal sequence, followed by a trypsin-like serine protease domain and one or two PDZ modules at the C-terminus, which permit protein–protein interactions [23, 27–29]. Inactivation of the *htr*A gene by mutation regularly results in high temperature sensitivity of many bacteria [30–35]. For a long time it was supposed that HtrA proteases are strictly functioning only inside the bacterial periplasm. However, we have previously introduced a new characteristic for the HtrAs of *Campylobacter jejuni* and *H. pylori.* These HtrA proteins can be actively secreted into the extracellular environment, where they cleave host cell factors [36–41]. It has been demonstrated that secreted HtrA from both species can open the adherens junctions in cultured polarized epithelial cells in vitro by cleaving the extracellular NTF (N-terminal fragment)-domain of E-cadherin, a well-known cell-to-cell adhesion factor [37, 39, 42]. Inactivation of *C. jejuni htrA* results in downregulated E-cadherin cleavage and bacterial transmigration across polarized cell monolayers in vitro [35, 39], and reduced apoptosis and immunopathology in the gut of infected mice in vivo [43, 44]. Similarly, HtrA is fundamental for the virulence of various other pathogens including *Yersinia enterocolitica*, *Klebsiella pneumoniae, Chlamydia trachomatis*, *Salmonella enterica*, *Listeria monocytogenes, Legionella pneumophila, Shigella flexneri, Burkholderia cenocepacia* and *Borrelia burgdorferi* [31, 32, 34, 45–50]. However, an *htrA* knockout strain in *H. pylori* is not yet available because the generation of mutants was unsuccessful in a broad collection of worldwide strains, suggesting that *htrA* may represent an essential gene in *H. pylori* [51, 52]. To study the role of HtrA further, we aimed to overexpress HtrA in *H. pylori* and examine various virulence properties of the bacteria. Our results show that overexpression of HtrA in *H. pylori* results in elevated secretion rates of the protease, cleavage of E-cadherin, bacterial transmigration and delivery of CagA into polarized epithelial cells.

## Results and discussion

### Introduction and expression of a second *htrA* gene copy in *H. pylori*

**Fig. 1:**
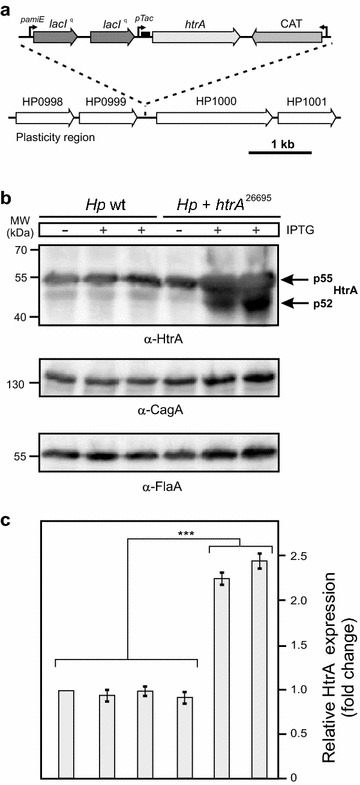
Chromosomal introduction of a second, inducible *htrA* gene copy in *H. pylori.* **a** Schematic presentation of the IPTG-inducible *htrA* ^26695^ expression construct with the chloramphenicol acetyl-transferase (CAT) cassette (*top*), which was cloned into the plasticity region of *H. pylori* between genes HP0999 and HP1000 (*bottom*). **b** This construct was transformed into *H. pylori* wild-type (*Hp* wt) strains 26695 and P12, and the expression of HtrA was investigated after 24 h growth in BHI medium in the presence or absence of 1 or 2 mM IPTG, respectively. The results for strain 26695 are shown. Western blotting using α-FlaA and α-CagA antibodies served as controls, indicating that equal amounts of proteins are present in the samples. **c** The band intensities of HtrA proteins were quantified densitometrically. The relative protein expression is given in “fold change”

*Helicobacter pylori htrA* is an essential bifunctional gene with crucial intracellular and extracellular functions [51, 52]. In order to study the function of *htrA* in more detail, we aimed to overexpress the protein by introduction of a second *htrA* gene copy of strain 26695 (*htrA* ^26695^) in the chromosome of *H. pylori*. For this purpose, we placed *htrA* ^26695^ under an inducible isopropyl-β-d-thiogalactopyranoside (IPTG)-responsive promotor as described [53]. Expression of *htrA* ^26695^ was driven by the p*Tac* promotor construct (Fig. 1a, top) derived from plasmid pILL2150 [53]. Promoter activity was described as tightly regulated for LacZ expression and suitable for the analysis of essential *H. pylori* genes [53]. We transformed this construct in two *H. pylori* wild-type strains, P12 or 26695 (called *Hp* wt) and the resulting transformants were designated *Hp htrA* ^26695^. We obtained similar results for both *H. pylori* strains and subsequently show the representative results for one set of experiments. *Hp* wt and *Hp htrA* ^26695^ were grown for 24 h in brain heart infusion (BHI) liquid broth medium containing 10% FCS in the presence or absence of 1 mM or 2 mM IPTG, respectively, and the resulting lysates were checked for expression of HtrA and other well-known *H. pylori* proteins using Western blotting. The results indicate that HtrA expression in *Hp htrA* ^26695^ in the presence of IPTG increased up to about 2.4-fold compared to the control without IPTG, but did not change in *Hp* wt (Fig. 1b, c). Control blots using α-CagA and α-FlaA antibodies showed that the expression of CagA and FlaA proteins remained stable over time and were unaffected by addition of IPTG to all strains (Fig. 1b). These results demonstrate that the IPTG-dependent expression system works well for *htrA* ^26695^ in two *H. pylori* strains and is very useful for further analyses.

### Overexpression of HtrA in *H. pylori* enhances its proteolytic activity

**Fig. 2:**
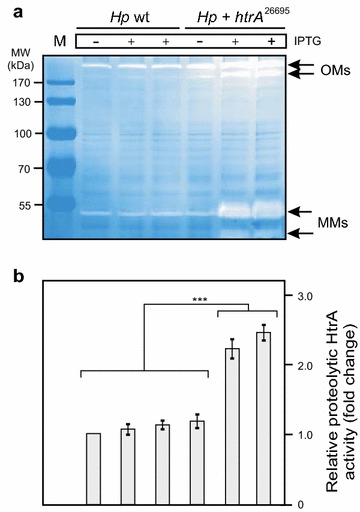
Expression of two *htrA* gene copies in *H. pylori* enhances the overall proteolytic activity of HtrA. **a** *H. pylori* wild-type strain 26695 (*Hp* wt) and 26695 transformed with *htrA* ^26695^ were grown for 24 h in BHI broth medium in the presence or absence of 1 or 2 mM IPTG, respectively. Bacterial pellets were prepared and subjected to investigation of protease activity by casein zymography. The position of proteolytically active HtrA monomers (MMs) and oligomers (OMs) is indicated with *arrows*. M, protein size marker. **b** The band intensities of proteolytically active HtrA proteins were quantified densitometrically. The relative HtrA activity is given in “fold change”

Next, we aimed to analyse if *Hp* wt or *Hp htrA* ^26695^ can form proteolytically active HtrA oligomers in the absence or presence of IPTG. For this purpose, the samples generated for Fig. 1b were subjected to casein zymography. Bacterial pellets were loaded onto 0.1% casein containing gels and separated under non-reducing conditions and then renatured as described [38]. The results show that HtrA activity of *Hp htrA* ^26695^ increased up to ~2.5-fold in the presence of IPTG compared to *Hp* wt or the control without IPTG, giving rise to active HtrA oligomers with a molecular weight ranging from ~180 kDa to more than 200 kDa in the cell pellet (Fig. 2a, arrows). As further control, corresponding signals for proteolytically active HtrA were at a similar basal level in both strains in the absence of IPTG (Fig. 2a, b).

### Induction of HtrA leads to higher secretion levels of HtrA, but not VacA and GGT

**Fig. 3:**
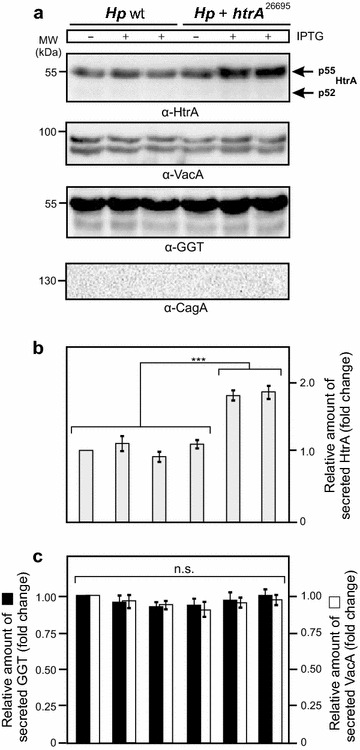
Elevated HtrA secretion levels in *H. pylori* does not affect the extracellular delivery of VacA and GGT. **a** *H. pylori* wild-type strain 26695 (*Hp* wt) and 26695 transformed with *htrA* ^26695^ were grown for 24 h in BHI broth medium with 1% β-cyclodextrin in the presence or absence of 1 or 2 mM IPTG, respectively. Bacteria-free supernatants were prepared and subjected to Western blotting using α-HtrA, α-VacA and α-GGT antibodies. The blots against α-CagA served as control. The band intensities of secreted HtrA (**b**) as well as VacA and GGT (**c**) were quantified densitometrically. The relative protein expression is given in “fold change”. All secretion assays were done in triplicates

The next objective was to evaluate the level of secreted HtrA in the culture supernatants. After 24 h of growth, bacteria-free supernatants and cell pellets were prepared and the presence of secreted HtrA proteins in the supernatants was investigated by immunoblotting using α-HtrA antibodies (Fig. 3a). The results show that the bands for secreted HtrA in *Hp htrA* ^26695^ in the presence of IPTG increased up to ~1.8-fold compared to the strain without IPTG, but did not change significantly in *Hp* wt (Fig. 3a, b). As control, corresponding signals for secreted HtrA were at a similar basal level in both strains in the absence of IPTG (Fig. 3a, b). In further experiments, the supernatants were probed for two other well-known secreted *H. pylori* proteins, VacA and GGT. As shown in Fig. 3a, the band intensities for secreted VacA and GGT were constantly stable in both strains and did not change by adding IPTG (Fig. 3a, c). On the other hand, CagA is a well-known translocated T4SS effector protein, not secreted into the supernatant [54]. The α-CagA blots of the supernatants are devoid of CagA, indicating absence of lysed bacteria and cell debris in our samples as expected (Fig. 3a). Taken together, these experiments demonstrate that secretion of HtrA by *Hp htrA* ^26695^ is significantly enhanced after addition of IPTG compared to the *Hp* wt control, while the secretion levels of VacA and GGT remained unaffected.

### Overexpression of HtrA does not affect host cell binding and IL-8 secretion by *H. pylori*

**Fig. 4:**
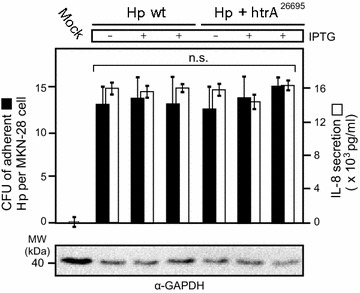
Higher expression of HtrA does not change host cell binding capabilities and IL-8 production by *H. pylori.* IPTG-induced or control *H. pylori* wild-type strain 26695 (*Hp* wt) and 26695 transformed with *htrA* ^26695^ were grown for 24 h in BHI broth medium with 10% FCS. Polarized MKN-28 cells were then co-incubated with *H. pylori* for 8 h using an MOI of 50. The numbers of adherent bacteria were determined by adhesion assays and counting the CFU on GC agar plates. The concentrations of secreted chemokine IL-8 were quantified by standard ELISA. All infection assays were done in triplicates

As next, we aimed to study the functional role of HtrA overexpression during infection of epithelial cells. For this purpose, monolayers of polarized MKN-28 cells were infected for 8 h with IPTG-induced or control *Hp* wt and *Hp htrA* ^26695^, respectively. To test if differential HtrA expression might affect host cell binding by *H. pylori*, we determined the CFU of bound bacteria by an established protocol [55]. The results show that the number of bound bacteria was similar between the samples and varied only between 10 and 16 CFU per MKN-28 cell (Fig. 4). In addition, we have analyzed the amount of chemokine IL-8 secreted into the supernatants. The levels of IL-8 were also at similar high level between the samples and varied only between ~12,000 and 17,000 pg/mL. These results suggest that overexpression of HtrA by IPTG induction does not affect the bacteria’s viability and host cell binding capabilities. The T4SS-dependent activity of *H. pylori* towards IL-8 secretion was also very high and similar between the samples, suggesting that T4SS functions are intact and remain unchanged with regard to the pro-inflammatory responses during infection with the different strains.

### Overexpression of HtrA enhances disruption of cell-to-cell junctions by *H. pylori*

**Fig. 5:**
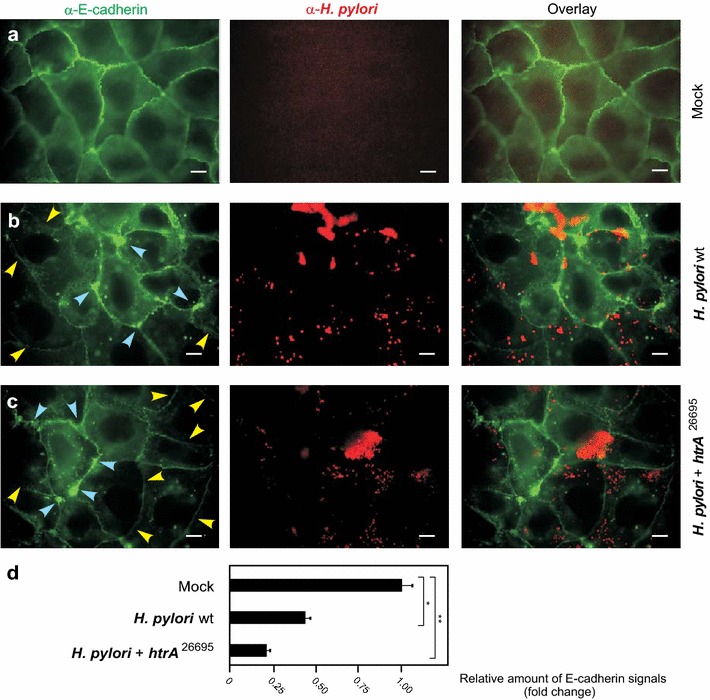
Enhanced disruption of cell-to-cell junctions by *H. pylori* expressing two *htrA* gene copies. Polarized Caco-2 cells were left untreated (mock) (**a**) or infected for 24 h with *H. pylori* wild-type (wt) (**b**) and *H. pylori htrA* ^26695^ (**c**). The cells were fixed with methanol and subjected to immunofluorescence using α-E-cadherin (*green*) and α-*H. pylori* (*red*) antibodies. *Arrowheads* mark cells showing significantly downregulated (*blue*) or disrupted (*yellow*) E-cadherin signals. *Scale bars*, 10 μm. **d** Quantification of overall fluorescence signals for E-cadherin stainings, which are given in “fold change”. The total E-cadherin signal in the uninfected mock control was set as “1”

In the next set of experiments, confluent polarized Caco-2 cells were infected with the various IPTG-induced *H. pylori* strains for 24 h and subsequently fixed for immunofluorescence microscopy staining against the adherens junction protein E-cadherin and *H. pylori*. The results confirm that the signals of *Hp* wt or *Hp htrA* ^26695^ bacteria (red) attached to the host cells are similarly high between the samples. However, while the mock control cells exhibited typical E-cadherin signals between all neighbouring cells, *H. pylori* infection disrupted the E-cadherin staining significantly (Fig. 5b–d). Individual cells showing downregulated or dislocated E-cadherin signals are marked with blue and yellow arrowheads, respectively (Fig. 5b, c). The number of cells with changed E-cadherin patterns was more pronounced during infection with *Hp htrA* ^26695^ (Fig. 5c, d). Longer infection times up to 48 h, however, led to a complete disruption of the E-cadherin patterns by *Hp htrA* ^26695^ bacteria (data not shown). These data indicate that overexpression of HtrA is associated with enhanced damage of the cell-to-cell junctions over time.

### Overexpression of HtrA enhances bacterial transmigration across polarized cells

**Fig. 6:**
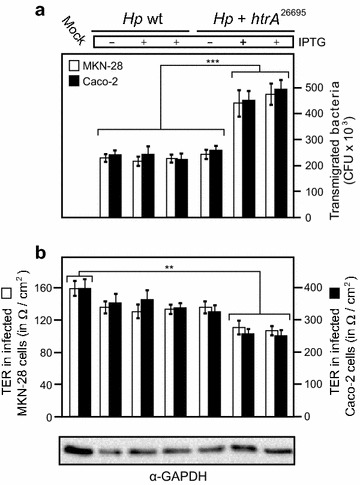
Upregulation of bacterial transmigration by *H. pylori* expressing two *htrA* genes. **a** Caco-2 and MKN-28 cells were cultured in a transwell filter system for 14 days to reach polarized monolayers, followed by apical infection with IPTG-induced or control *Hp* wt or *Hp htrA* ^26695^, respectively. After 24 h, transmigrated bacteria in the bottom chambers were quantified by counting CFUs. The results show that the number of transmigrated *Hp htrA* ^26695^ bacteria in the presence if IPTG increased ~2.5-fold compared to IPTG-induced control *Hp* wt. **b** Determination of the TER values in the same set of experiments. All experiments were done in triplicates

In addition, we determined the transmigration rates by the different *H. pylori* strains. For this purpose, polarized Caco-2 and MKN-28 cells were grown in a transwell filter system for 14 days to reach confluent monolayers. The cells were infected with IPTG-induced or control *Hp* wt or *Hp htrA* ^26695^ for 24 h in the apical chamber. Transmigrated bacteria were harvested from the bottom chambers, grown on GC agar plates, and the CFUs were determined (Fig. 6a). The results show that the number of transmigrated *Hp htrA* ^26695^ bacteria in the presence of IPTG were about 420–520 × 10^3^ CFU and increased up to ~2.2-fold compared to the corresponding IPTG-induced control *Hp* wt bacteria (Fig. 6). As a further control, the numbers of transmigrated bacteria of both strains in the absence of IPTG were at a similar basal level of approximately 200–265 × 10^3^ *H. pylori* (Fig. 6a). In addition, we measured the transepithelial electrical resistance (TER) in the same experiments. In agreement with the results obtained above, the data show that the TER values dropped down significantly (Fig. 6b), correlating with enhanced cell damage (Fig. 5) and transmigration by *Hp htrA* ^26695^ bacteria in the presence of IPTG (Fig. 6a).

### Overexpression of HtrA results in elevated E-cadherin cleavage and CagA phosphorylation

**Fig. 7:**
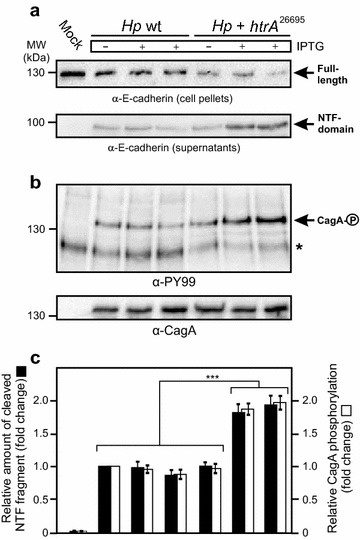
Elevated E-cadherin cleavage and CagA translocation in host cells by overexpression of HtrA. Polarized MKN-28 cells were infected with IPTG-induced or control *Hp* wt or *Hp htrA* ^26695^ for 24 h. **a** Cell pellets and supernatants were prepared and subjected to α-E-cadherin Western blotting. The full-length E-cadherin signals (130 kDa) in the cell pellets and cleaved-off NTF-domain (90 kDa) in the culture supernatants are marked with *arrows*. **b** Translocation and phosphorylation of CagA (*arrow*) were analyzed using α-PY-99 and α-CagA antibodies, respectively. The *asterisk* marks a phosphorylated 125 kDa host cell protein migrating below CagA. **c** The band intensities of cleaved E-cadherin NTF-fragments and phospho-CagA signals were quantified densitometrically. The relative amounts of NTF-fragment and phosphorylated CagA are given in “fold change”. All experiments were done in triplicates

As next we investigated the cleavage of E-cadherin in infected vs. non-infected polarized epithelial cell lines by Western blotting after 24 h of incubation (Fig. 7). The results show that the intensity of cell-associated full-length E-cadherin signals dropped-down during infection with *Hp htrA* ^26695^ bacteria in the presence of IPTG and the corresponding cleaved-off NTF-domain, present in the supernatant, increased up to ~twofold compared to the corresponding IPTG-induced control *Hp* wt bacteria (Fig. 7a, c). As another control, the signals of the NTF-fragment produced by both strains in the absence of IPTG were at a similar basal level (Fig. 7a, c). Remarkably, the extent of E-cadherin cleavage induced by the different strains correlated with the intensity of signals obtained for translocated phospho-CagA in the same experiments (Fig. 7b, c). The strongest phospho-CagA signals were always observed for *Hp htrA* ^26695^ bacteria in the presence of IPTG, suggesting that increased HtrA expression and activity significantly positively regulate T4SS functions with regard to translocation and phosphorylation of CagA in polarized epithelial cells.

## Conclusions

Diverse pathogens encode proteases with crucial functions during infection, but knowledge on secreted proteases and their activities in *H. pylori* is very limited. In many bacteria, HtrA is a well-recognized factor in the periplasm, which contains chaperone and proteolytic functions with important roles in protein quality control involved in stress tolerance and bacterial survival [23, 25–29]. In addition, it was demonstrated that HtrA has a significant impact on the virulence of multiple bacterial pathogens including *Borrelia, Burkholderia*, *Campylobacter, Chlamydia*, *Klebsiella, Legionella, Listeria, Salmonella*, *Shigella* and *Yersinia* species. Interestingly, *htrA* does not appear as an essential gene in each of these bacteria because Δ*htrA* knockout mutation has been described [31, 32, 34, 43–50]. In contrast, inactivation of the *htrA* gene in *H. pylori* has been unsuccessful in more than one hundred worldwide isolates, but the reasons for this failure are still unclear [37, 51, 52]. Remarkably, it was also demonstrated that pharmacological inhibition of HtrA protease activity effectively killed *H. pylori*, while it did not affect the growth and viability of other Gram-negative pathogens including *Salmonella* and *Shigella* [52].

Research progress on *H. pylori* HtrA is mainly hampered by the lack of Δ*htrA* knockout mutants. Thus, other genetic manipulation strategies are required to study HtrA function during the infection process. Here we developed a genetic approach to overexpress HtrA in two clinical isolates, P12 and 26695. For this purpose, a second *htrA* gene copy was introduced into the *H. pylori* chromosome and placed under an IPTG-inducible promotor [53]. Once the HtrA proteins are translated by the bacteria they are delivered into the periplasm and subsequently secreted into the extracellular environment. This important new aspect seems to be conserved among a wide range of worldwide *H. pylori* isolates [52]. We could show here that overexpression of HtrA enhanced not only its proteolytic activity by up to ~2.5-fold, but also the secretion of the protease by ~1.8-fold. Interestingly, the secretion of other well-known bacterial virulence determinants, VacA and GGT, was not affected by HtrA overexpression, suggesting that the secretion of these factors follow different, non-linked pathways. In addition, we could demonstrate that various virulence-associated properties of *H. pylori* were also not affected including bacterial attachment to the epithelial cells and induction of pro-inflammatory responses such as the secretion of IL-8. In contrast, the transepithelial migration of *H. pylori* overexpressing HtrA increasing significantly up to ~2.2-fold compared to the control bacteria. This phenotype was accompanied by significantly enhanced damage to the adherens junction protein E-cadherin. Our Western blotting data demonstrated that HtrA-mediated cleavage of full-length E-cadherin was enhanced, leading to elevated levels of the 90 kDa E-cadherin NTF-fragment in the supernatants of infected cells. Immunofluorescence microscopy confirmed these observations and showed that the cell-to-cell junctions of infected Caco-2 cells were significantly more disrupted after 24 h compared to the wild-type control infection, explaining why higher numbers of bacteria can cross the epithelial barrier and reach basolateral compartments. Finally, we observed that the levels of CagA translocation and phosphorylation increased up to ~twofold in HtrA-overexpressing *H. pylori* compared to the control bacteria. These observations can be explained by reports showing that CagA delivery into host cells requires a receptor, which was identified as the basolateral integrin member α_5_β_1_ [56–62]. Integrins are well-known mammalian cell adhesion receptors, which facilitate anchoring of host cells to the extracellular matrix and which are absent at apical surfaces [63, 64]. These findings let us to suggest a novel mechanism how the T4SS of *H. pylori* works in polarized epithelial cells by cooperating with the secreted serine protease HtrA, which opens cell-to-cell junctions. Using an inducible genetic system to overexpress HtrA, we could enhance the proteolytic activity of HtrA, necessary for elevated paracellular transmigration of *H. pylori* across the polarized epithelial cells to reach basolateral membranes and inject CagA in an integrin-dependent fashion. Extensive research has shown in recent years that the above discussed features basically resemble a phenotype, called epithelial-mesenchymal transition (EMT). Gastric cancerogenesis is known for its aggressiveness and tendency to metastasize. EMT is the initial step in metastasis, orchestrated by various cellular factors [65]. We proposed that the activity of secreted HtrA is maybe the initial step in a signaling cascade, followed by CagA and probably others, that triggers EMT in gastric epithelial cells. Translocated CagA can then deregulate cell polarity and scattering, by various pathways including the interaction with partioning kinase Par1b changing cell polarity [66] and by stabilizing Snail, a transcriptional repressor of E-cadherin expression [67]. Taken together, these data provide for the first time genetic evidence that HtrA is a major novel virulence factor of *H. pylori*, controlling multiple pathogenic activities of this important microbe.

## Methods

### MKN-28 and Caco-2 cell culture and *H. pylori* infection

Human MKN-28 cells (JCRB, #0253) were originally isolated from gastric adenocarcinoma. The Caco-2 cells (ATCC HTB-37) were obtained from a human colon adenocarcinoma. Both cell lines have been extensively used over the last twenty years as models for studying the gastrointestinal barrier. Cells were cultured in 6-well plates with RPMI1640 or DMEM medium, respectively, containing 4 mM glutamine (Invitrogen, Karlsruhe/Germany), and 10% FCS (Invitrogen, Karlsruhe/Germany) [68]. *H. pylori* strains 26695, P12 and their mutants were grown on horse serum GC agar plates supplemented with nystatin (1 μg/mL), vancomycin (10 μg/mL) and trimethoprim (5 μg/mL), and if necessary with 4 μg/chloramphenicol per mL. Growth was performed for 2 days at 37 °C in anaerobic chambers containing a CampyGen gas mix (Oxoid, Wesel/Germany) at 37 °C [69]. *H. pylori* was harvested and resuspended in phosphate buffered saline (PBS, pH 7.4) using sterile cotton swabs (Carl Roth, Karlsruhe/Germany). The bacterial concentration was measured in a spectrophotometer as optical density (OD) at 600 nm (Eppendorf, Hamburg/Germany). Infections were carried out at a multiplicity of infection (MOI) of 50 [70]. All infection assays were done in triplicates.

### *H. pylori* mutagenesis

To introduce a second *htrA* gene copy in the *H. pylori* chromosome, we made use of the previously generated IPTG-inducible LacI^q^ p*Tac* system for *lacZ* gene expression as cloned in vector pILL2150 [53]. In this system, the promoters were engineered to be under the control of *H. pylori* RNA polymerase. The *amiE* gene promoter of *H. pylori* was taken to constitutively express the LacI^q^ repressor, which is present in two copies (Fig. 1a, top). Expression of the *lacZ* reporter gene was driven by the p*Tac* promotor as described [53]. We replaced the *lacZ* gene of pILL2150 by the *htrA* gene of strain 26695 using the *Nde*I and *Bam*HI restriction sites. Then the complete cassette shown in Fig. 1a (top) was introduced in the chromosomal plasticity region of *H. pylori* strains P12 and 26695 (between ORFs HP0999 and HP1000) as shown in Fig. 1a (bottom) using established transformation methods [71, 72]. At the 3′ end, a chloramphenicol resistance gene cassette (CAT) was added to select clones. The correct integration and expression of the *htrA* gene was verified by PCR and Western blotting, respectively.

### HtrA, VacA and GGT secretion assays

Wild-type and mutant *H. pylori* strains were suspended in BHI medium supplemented with 1% β-cyclodextrin (Sigma Aldrich) [73]. The optical density was determined and adjusted to OD_600_ = 0.2. To allow bacterial protein secretion in the culture supernatant, *H. pylori* was grown for 24 h under shaking at 160 rpm in the presence or absence of IPTG (Sigma Aldrich). The cell pellets and the supernatants were prepared by centrifugation at 4000 rpm. The supernatants were transferred through 0.21 μm sterile filters (Sigma Aldrich) to remove remnant bacterial cells. Lack of live bacteria in the supernatant was verified by the absence of bacterial growth after 5 days of incubation on GC agar plates. The resulting bacterial pellets and supernatants were then analysed by Western blotting as described below.

### Transwell infection studies

MKN-28 and Caco-2 cells were cultured on 0.33 cm^2^ cell culture inserts with 3 μm pore size (Corning Life Sciences, Schiphol/Netherlands). The cells were grown to confluent monolayers, and then incubated for another 14 days to allow cell polarization [39]. TER was measured with an Electrical Resistance System (ERS) (World Precision Instruments, Berlin/Germany). Maximal TER values indicated that the monolayers reached maximal cell polarity [39]. The cells were infected in the apical compartment at MOI of 50 and the numbers of transmigrated bacteria were quantified in aliquots taken from the basal chambers and counting colony forming units (CFU) on GC agar plates after 5 days of incubation [35].

### Cell binding assay

Infection of MKN-28 and Caco-2 cell monolayers was performed at a density of 3.5 × 10^5^ cells in 6-well plates as described previously [55]. After infection, infected cells were washed three times with 1 mL of pre-warmed culture medium per well to remove non-adherent bacteria. To determine the total CFU corresponding to cell-associated bacteria, the infected monolayers were incubated with 1 mL of 0.1% saponin in PBS at 37 °C for 15 min. The resulting suspensions were diluted and plated on GC agar plates. The CFUs were counted after 5 days of incubation.

### Casein zymography

Undiluted aliquots of the bacteria were loaded onto 10% SDS–PAGE gels containing 0.1% casein (Carl Roth, Karlsruhe/Germany) and separated by electrophoresis under non-reducing conditions. After protein separation, the gel was renatured in 2.5% Triton X-100 solution at room temperature for 60 min with gentle agitation, equilibrated in developing buffer (50 mM Tris–HCl, pH 7.4, 200 mM NaCl, 5 mM CaCl_2_, 0.02% Brij35) at room temperature for 30 min with gentle agitation, and incubated overnight in fresh developing buffer at 37 °C. Transparent HtrA bands with caseinolytic activity were visualized by staining with 0.5% Coomassie Blue R250 as described [35, 39].

### Antibodies

The following antibodies were purchased: rabbit polyclonal α-CagA antibody (Austral Biologicals, San Ramon, CA/USA), monoclonal pan-phosphotyrosine α-PY99 (Santa Cruz, Santa Cruz, CA/USA), rabbit α-GAPDH (Santa Cruz), rabbit α-*H. pylori* (Dako, Glostrup/Denmark) and two monoclonal antibodies directed against the extracellular domain of E-cadherin, H-108 (Santa Cruz) and CD324 (BD Biosciences, San Jose, CA/USA). HtrA proteins were detected by rabbit polyclonal α-HtrA antiserum raised against purified recombinant HtrA (Biogenes, Berlin/Germany). Rabbit polyclonal α-FlaA and α-GGT antibodies were described previously [74, 75]. The α-VacA antibody (#123) was kindly provided by Timothy Cover (Nashville, TN/USA).

### Immunofluorescence staining and microscopy

Immunofluorescence staining with different antibodies as shown in each experiment was performed as described [76]. Briefly, cell samples were fixed with methanol at −20 °C for 10 min followed by permeabilization with 0.5% Triton-X100 for 1 min and blocking with 1% BSA, 0.1% Tween-20 in PBS for 30 min. Proteins were stained with the above mentioned α-E-cadherin (BD) and α-*H. pylori* antibodies. As secondary antibodies, we used TRITC (tetramethylrhodamine isothiocyanate)-conjugated goat anti-rabbit and FITC (fluorescein isothiocyanate)-conjugated goat anti-mouse (Thermo Fisher Scientific, Darmstadt/Germany). Samples were analysed using a Leica DMI4000B fluorescence microscope and different lasers (Leica Microsystems, Wetzlar/Germany). Images were obtained via LAS AF computer software **(**Leica Microsystems) and E-cadherin staining was quantified as “fold change” using the ImageJ Software (version 2.0). The mock control was set as “1”.

### SDS–PAGE, dot blots and immunoblotting

Bacterial pellets, cell-free supernatants or infected cells were mixed with equal amounts of 2× SDS–PAGE buffer and boiled for 5 min. Proteins were separated by SDS–PAGE on 8% polyacrylamide gels and blotted onto PVDF membranes (Immobilon-P, Merck Millipore) as described [77]. Before addition of the antibodies, membranes were blocked in TBST buffer (140 mM NaCl, 25 mM Tris–HCl pH 7.4, 0.1% Tween-20) with 3% BSA or 5% skim milk for 1 h at room temperature [78]. As secondary antibodies, horseradish peroxidase-conjugated α-mouse or α-rabbit polyvalent rabbit and pig immunoglobulin, respectively, were used (Life Technologies, Darmstadt/Germany). Antibody detection was performed with the ECL Plus chemiluminescence Western Blot kit (GE Healthcare Life Sciences, Munich/Germany) [79].

### Quantification of IL-8 chemokines by ELISA

MKN-28 cells were incubated for 8 h with *H. pylori*, and mock cells with medium served as negative control. The culture supernatants were collected and stored at −80 °C until assayed. IL-8 concentrations in the supernatants were determined by standard ELISA according to manufacturer’s procedures (Becton–Dickinson, Heidelberg/Germany) [80].

### Quantification of band intensities in Western blots and casein gels

Quantification of band signals on immunoblots was performed by densitometric analysis using the Image Lab software (BioRad, Munich/Germany) and indicated the “fold change” of protein expression or phosphorylation level per sample. As shown in the corresponding figures, the control band on each gel was set as “1”.

### Statistics

All data were evaluated via Student’s t test with SigmaPlot statistical software (version 13.0). Statistical significance was defined by *p* ≤ *0.05* (*), *p* ≤ *0.01* (**) and *p* ≤ *0.001* (***).
